# Reply to Wagman et al.: Data-driven assessments should establish the landscape of what is “within reach” of malaria transmission control

**DOI:** 10.1073/pnas.2211931119

**Published:** 2022-09-12

**Authors:** Carlo Costantini, Fabrice Chandre, Vincent Corbel, Nicolas Moiroux, Frédéric Simard, Claire Sangbakembi-Ngounou, Diego Ayala

**Affiliations:** ^a^MIVEGEC (Maladies infectieuses et vecteurs : écologie, génétique, évolution et contrôle), Université de Montpellier, CNRS, Institut de Recherche pour le Développement, Montpellier 34394, France;; ^b^Institut Pasteur de Dakar, BP 220 Dakar, Senegal;; ^c^Oswaldo Cruz Foundation, Rio de Janeiro 21040-900, Brazil;; ^d^Medical Entomology Laboratory, Institut Pasteur de Bangui, BP 923 Bangui, Central African Republic;; ^e^Medical Entomology Unit, Institut Pasteur de Madagascar, BP 1274, 101 Antananarivo, Madagascar

Commenting on results of Sangbakembi-Ngounou et al. (1), who showed extensive diurnal and outdoor biting by three of the most important Afrotropical malaria mosquitoes, Wagman et al. (2) aptly warn against the misconception that insecticide treated nets (ITNs) and indoor residual spraying (IRS)—currently the most efficient malaria vector control tools—are redundant, making the case for rethinking single intervention approaches in the face of budget constraints. The argument that indoor insecticidal interventions like IRS can be effective regardless of location or timing of mosquito blood-feeding is grounded on the resting behavior of malaria vectors: Reductions in transmission may be achieved despite diurnal or outdoor biting, provided that a significant fraction of the mosquito population rests on surfaces treated with effective insecticides (1, 2).

Sangbakembi-Ngounou et al. (1), however, further bring to attention dangers arising from ascertainment biases in mosquito biting behavior. Indeed, the unexpected finding of substantial diurnal biting resulted from their modification of conventional practices in malaria transmission assessment. Ascertainment biases in malaria vector resting behavior are potentially equally likely. For example, resolving uncertainties about the size (3) or nonuniform nature (4)—and, consequently, nonuniform exposure to indoor insecticides (5)—of the vector population resting outdoors, as well as filling data deficiencies about mosquito biting and resting in structures where people occur mostly during daytime (e.g., schools, offices, or public buildings—structures that are generally not covered by either IRS or ITNs), could help throw light on IRS variable control outcomes and, accordingly, on the limitations of IRS efficacy or cost-effectiveness under some circumstances, either deployed on its own (6) or in association with ITNs (7).

Other potential explanatory sources of variability arise from additional uncertainties: Despite efforts to investigate the preprandial behavior of indoor-biting malaria vectors, there are still incertitudes whether mosquitoes, once inside domestic households, rest on treated surfaces before coming to the host and biting, or the extent and impact of postprandial exposure to insecticides. Similarly, some insecticidal formulations deter to variable degrees mosquitoes from entering into or remaining inside treated structures, inhibit blood-feeding, or induce them to escape outdoors, but the precise role and consequences of these effects upon malaria transmission are scantily appreciated (8). Furthermore, we are just beginning to understand how genetic polymorphisms and insecticide resistances (i.e., physiological or behavioral) and their interactions with environmental modulators influence mosquito biting and resting and their effects on transmission (9).

Importantly, extrapolation of findings from individual studies aimed at setting continent-wide control policies may also lead to unpredictable outcomes in the face of heterogeneities due to, *inter alia*, the genetically much diversified (10) and constantly evolving (5, 11) communities of vectors. Thus, undogmatic, data-driven, unbiased quantitative empirical approaches, informed by and feeding back into theoretical models (8), are necessary to assess the consequences of epidemiologically relevant behaviors of local vector communities, to understand the heterogenous and volatile landscape of what may be “out of control” or otherwise “within reach” of current interventions, with the goal of sustainable control of residual malaria transmission by complementary tools whenever needed.
